# Association of Genetically Predicted Insomnia With Risk of Sepsis: A Mendelian Randomization Study

**DOI:** 10.1001/jamapsychiatry.2023.2717

**Published:** 2023-08-09

**Authors:** Marianne S. Thorkildsen, Lise T. Gustad, Randi M. Mohus, Stephen Burgess, Tom I. L. Nilsen, Jan K. Damås, Tormod Rogne

**Affiliations:** 1 Gemini Center for Sepsis Research at Institute of Circulation and Medical Imaging, Norwegian University of Science and Technology, Trondheim, Norway; 2 Faculty of Nursing and Health Sciences, Nord University, Levanger, Norway; 3 Department of Medicine and Rehabilitation, Levanger Hospital, Nord-Trøndelag Hospital Trust, Levanger, Norway; 4 Clinic of Anesthesia and Intensive Care, St Olavs Hospital, Trondheim, Norway; 5 MRC Biostatistics Unit, University of Cambridge, Cambridge, United Kingdom; 6 Cardiovascular Epidemiology Unit, Department of Public Health and Primary Care, University of Cambridge, Cambridge, United Kingdom; 7 Department of Circulation and Medical Imaging, Norwegian University of Science and Technology, Trondheim, Norway; 8 Department of Public Health and Nursing, Norwegian University of Science and Technology, Trondheim, Norway; 9 Centre of Molecular Inflammation Research, Norwegian University of Science and Technology, Trondheim, Norway; 10 Department of Clinical and Molecular Medicine, Norwegian University of Science and Technology, Trondheim, Norway; 11 Department of Infectious Diseases, Clinic of Medicine, St Olavs Hospital, Trondheim, Norway; 12 Department of Chronic Disease Epidemiology, Yale University School of Public Health, New Haven, Connecticut

## Abstract

**Importance:**

Insomnia has been associated with altered inflammatory response as well as increased risk of infections and sepsis in observational studies. However, these studies are prone to bias, such as residual confounding. To further understand the potential causal association between insomnia and sepsis risk, a 2-sample Mendelian randomization (MR) approach should be explored.

**Objective:**

To evaluate whether genetically predicted insomnia is associated with risk of sepsis.

**Design, Setting, and Participants:**

Two-sample MR was performed to estimate the association between genetically predicted insomnia and sepsis risk. Data were obtained from a genome-wide association study identifying 555 independent genetic variants (*R*^2^ < 0.01) strongly associated with insomnia (*P* < 5 × 10^−8^). Sensitivity analyses were conducted to address bias due to pleiotropy and sample overlap, along with mediation analyses and sex-stratified analyses. The insomnia data set included 2.4 million individuals of European ancestry from the UK Biobank and 23andMe. For sepsis, 462 918 individuals of European ancestry from the UK Biobank were included. Data were extracted between February and December 2022 and analyzed between March 2022 and March 2023.

**Exposure:**

Genetically predicted insomnia.

**Main Outcome and Measure:**

Sepsis.

**Results:**

There were 593 724 individuals with insomnia and 10 154 cases of sepsis. A doubling in the population prevalence of genetically predicted insomnia was associated with an odds ratio of 1.37 (95% CI, 1.19-1.57; *P* = 7.6 × 10^−6^) for sepsis. Sensitivity analyses supported this observation. One-third of the association between genetically predicted insomnia and risk of sepsis was mediated through a combination of cardiometabolic risk factors for sepsis (body mass index, type 2 diabetes, smoking, or cardiovascular disease; overall proportion, 35.2%; 95% CI, 5.1-76.9). The association between insomnia and sepsis was more pronounced among women compared with men (women: odds ratio, 1.44; 95% CI, 1.24-1.68; men: OR, 1.10; 95% CI, 0.86-1.40).

**Conclusions and Relevance:**

The concordance between these findings and previous observational studies supports that insomnia is potentially causally associated with the risk of sepsis. Thus, insomnia is a potential preventable risk factor of sepsis that should be further investigated, also in non-European populations.

## Key Points

**Question:**

Is genetically predicted insomnia associated with risk of sepsis?

**Findings:**

In this mendelian randomization study including 593 724 individuals, a doubling in the genetically predicted prevalence of insomnia was significantly associated with a 37% increased risk of insomnia.

**Meaning:**

These findings support a potential causal association between insomnia and the risk of sepsis.

## Teaser

This mendelian randomization study evaluates whether genetically predicted insomnia is associated with risk of sepsis.

## Introduction

Insomnia is the most common sleep disorder and is associated with numerous adverse health outcomes.^1^ In particular, insomnia is associated with an altered immune function and elevated systemic levels of inflammatory markers.^2^ Evidence also suggests that sleep deprivation may impact inflammatory activation more in women than in men.^3^ The severity and outcome of an infection is closely linked to the ability of the immune system to efficiently eradicate pathogens without harming the host. In the case of a dysregulated immune response, sepsis can develop, with accompanying high morbidity and mortality.^4^

A recent observational study reported that insomnia increased the risk of bloodstream infection, a condition closely linked to sepsis.^5^ Importantly, since insomnia and systemic infections may share common causes, the observed association between insomnia and infectious disease risk could be biased due to residual confounding. Mendelian randomization (MR) is a method that uses genetic variants as instruments for modifiable risk factors to reduce the influence of confounding and reverse causation.^6^ This approach mimics a randomized clinical trial by using the principle of random allocation of genetic variants.

Our aim was to assess if insomnia is associated with risk of sepsis when applying instrumental variable analyses using genetic instruments. We also aimed to estimate the proportion of the association between genetically predicted insomnia and sepsis that is mediated through known cardiometabolic risk factors of sepsis, ie, body mass index (BMI), smoking, type 2 diabetes (T2D), and cardiovascular disease (CVD).

## Method

We used a 2-sample MR approach, following the Strengthening the Reporting of Observational Studies in Epidemiology (STROBE) reporting guideline for MR. Single-nucleotide variants (SNVs) were used as genetic instruments, and for each SNV, we calculated the Wald ratio, defined as the SNV-outcome association divided by the SNV-exposure association.^6^ For an instrument to be valid, it must be associated with the exposure, cannot be associated with any confounder of the exposure-outcome association, and cannot affect the outcome other than through the exposure.^6^

Only individuals of European ancestry were included in this study, as other ancestry groups were not available for all traits of interest, and a mix of ancestries can induce confounding due to genetic population structure.^6^ We obtained 555 genetic variants that were strongly associated (*P* < 5 × 10^−8^) with insomnia and that were independent of one another (*R* ^2^ <0.01) from a genome-wide association study (GWAS) evaluating 2.4 million individuals from the UK Biobank and 23andMe (Table; Supplement 1).^7^ For the multivariable MR analyses, we extracted genetic variants from relevant GWASs of BMI,^8^ T2D,^10^ smoking status,^9^ and CVD.^11^

**Table.** Overview of Genome-Wide Association Studies Used

Trait	Study	Cohorts	Phenotype definition	Population
Exposure				
Insomnia	Watanabe et al,^7^ 2022	UK Biobank and 23andMe	Cases: self-reported trouble falling asleep or self-reported diagnosis of insomnia Controls: not identified as cases (but had answered the questions)	Cases: 593 724; female, 390 751; male, 222 753 Controls: 1 771 286; female, 1 018 386; male, 993 280
Covariate				
Body mass index	Pulit et al,^8^ 2019	30 Cohorts, including UK Biobank	Measured body mass index at study participation	806 834
Smoking initiation	Liu et al,^9^ 2019	26 Cohorts, including deCODE and UK Biobank	Cases: self-reported smoking behavior Controls: never smoker	Cases: 557 337 Controls: 674 754
T2D	Mahajan et al,^10^ 2018	32 Cohorts, including 23andMe, deCODE and UK Biobank	Cases: T2D status based on a combination of diagnostic testing (fasting glucose or HbA_1c_), recorded diagnosis codes, or self-report Controls: not diagnosed with T2D	Cases: 74 124 Controls: 824 006
Cardiovascular disease	Kurki et al,^11^ 2023	FinnGen release 8	Cases: FinnGen code FG_CVD Controls: not classified as case	Cases: 174 499 Controls: 168 000
Outcome				
Sepsis (main analysis)	Ponsford et al,^12^ 2020	UK Biobank	Cases: explicit sepsis diagnosis codes Controls: not classified as case	Cases: 10 154 Controls: 452 764
Sepsis (sensitivity analysis)	Kurki et al,^11^ 2023	FinnGen release 8	Cases: FinnGen code AB1_OTHER_SEPSIS Controls: not classified as case	Cases: 10 666 Controls: 303 314

Abbreviations: HbA_1c_, hemoglobin A_1c_; T2D, type 2 diabetes.

Genetic associations for sepsis were extracted from a GWAS in the UK Biobank including 10 154 sepsis cases and 452 764 controls (maximum 16% overlap with insomnia GWAS).^12^ Cases were defined according to the explicit sepsis criteria defined in the most recent Global Burden of Disease Study of Sepsis.^4^ To validate our findings, and because sample overlap between the exposure and outcome GWASs may bias the estimate toward the confounded estimate, we included a sensitivity analysis of sepsis (*International Statistical Classification of Diseases and Related Health Problems, Tenth Revision *code A41) in FinnGen (Table).^11^

In the main analysis, using the inverse-variance weighted (IVW) method, we calculated the combined association across the Wald ratios for all SNVs, putting more emphasis to the SNVs with the lowest variance. For the IVW estimate to be unbiased, all included instruments must be valid. Thus, we conducted sensitivity analyses using the weighted median, weighted mode, and MR-Egger regression that provide unbiased results even in the presence of some invalid instruments (eg, due to pleiotropy) but at the cost of lower statistical power.^6^

We evaluated the proportion of the association between insomnia and sepsis that was mediated through 4 strong cardiometabolic risk factors of sepsis: BMI, T2D, smoking, and CVD.^8,9,10,11^ Using the SNVs identified as genetic instruments for insomnia, we calculated the direct association between insomnia and sepsis by conducting multivariable MR analyses with each of the 4 potential mediators at a time and then all mediators combined. The univariable analyses yielded the total association of genetically predicted insomnia. The proportion mediated was calculated as the direct association divided by the total association and subtracted from 1, and the SEs were estimated using bootstrapping.^13^ Lastly, to assess possible effect modification by sex, we performed univariable IVW analysis using sex-specific estimates of genetic associations for insomnia with the same SNV-selection criteria as for the main analysis.

We used R version 4.0.5 (The R Foundation) for data formatting and the TwoSampleMR package version 0.5.6 and MendelianRandomization version 0.7.0 packages in R for all analyses (eAppendix in Supplement 2).^14^ All estimates were multiplied by 0.693 ( = ln 2) to present the results as per doubling in the prevalence of insomnia. All data used in this study were retrieved from studies that had sought informed consent from their study participants. We only used deidentified, publicly available, summary-level data, which does not constitute human subjects research per 45 CFR 46.102 and therefore did not require institutional review board approval.

## Results

**Figure 1.**
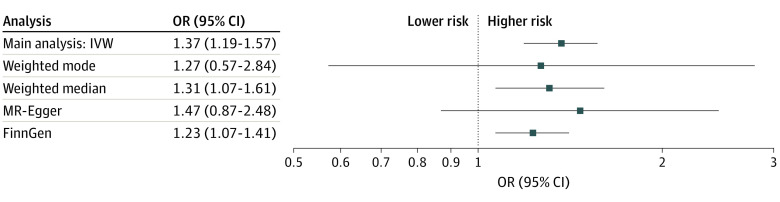
Mendelian Randomization (MR) Analyses of the Association Between Genetically Predicted Insomnia and Risk of Sepsis Odds ratios (ORs) with 95% CIs of risk of sepsis per genetically predicted doubling of the prevalence of insomnia. The FinnGen analysis used data from FinnGen instead of UK Biobank and was analyzed using inverse-variance weighting.

**Figure 2.**
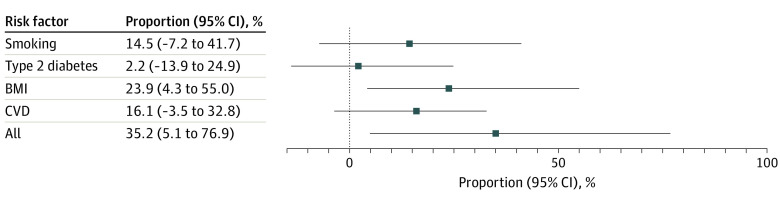
Proportion of the Association of Genetically Predicted Insomnia With the Risk of Sepsis Mediated Through Cardiometabolic Risk Factors BMI indicates body mass index; CVD, cardiovascular disease.

There were 593 724 individuals with insomnia and 10 154 cases of sepsis. The genetic variants used in the main analysis explained 3.9% of the variance of insomnia. A genetically predicted doubling in the prevalence of insomnia was associated with an odds ratio (OR) for sepsis of 1.37 (95% CI, 1.19-1.57; *P* = 7.6 × 10^−6^) (Figure 1). The weighted mode, weighted median, and MR-Egger analyses all supported the main analysis. The main analysis was also supported by the analysis using sepsis data from FinnGen. One-third of the association between genetically predicted insomnia and risk of sepsis was mediated through BMI, T2D, smoking, or CVD (Figure 2) but with a likely direct association independent of these factors (OR, 1.23; 95% CI, 1.03-1.45; *P* = .02). Considering each cardiometabolic risk factor separately, only BMI significantly mediated the association between genetically predicted insomnia and risk of sepsis. Finally, the harmful consequence of insomnia was more pronounced among women compared with men (women: OR, 1.44; 95% CI, 1.24-1.68; *P* = 2.6 × 10^−6^; men: OR, 1.10; 95% CI, 0.86-1.40; *P* = .44).

## Discussion

Our findings support a potential causal association between genetically predicted insomnia and risk of sepsis. This is in line with previous research and results from observational data, reporting that insomnia increases the risk of altered immune response^2,15^ and bloodstream infection.^5^ We observed that much of the association between genetically predicted insomnia and risk of sepsis was mediated through BMI, T2D, CVD, or smoking. This is supported by studies reporting that these factors are potentially caused or worsened by insomnia^15^ and are also potential causes of sepsis.^12^ However, most of the association between insomnia and risk of sepsis was not explained by these factors, indicating that insomnia may have a substantial direct influence on sepsis risk.

Numerous studies have reported that insomnia alters immune function through various mechanisms, suggesting a link to immune and inflammatory dysregulation and thus also to elevated sepsis risk.^2,15^ Interestingly, sleep deprivation is reported to cause a stronger inflammatory activation in women than men, which may explain the stronger effect of insomnia on sepsis risk in women than men observed in our study.^3^

### Strengths and Limitations

Our findings are strengthened by the consistent results across sensitivity analyses robust to pleiotropy and the evaluation in a separate outcome cohort with no overlap with the exposure GWAS. An important limitation to our study is that it only included individuals of European ancestry, and we encourage future studies to evaluate whether our findings replicate in other ancestry groups.

## Conclusions

The findings from this MR study are in accordance with those of previous observational studies and support a potential causal association between insomnia and risk of sepsis.

## Notes

Supplement 1. Single-Nucleotide Polymorphism Associations Used in Main Analysis

Supplement 2. **eAppendix.** Example Code for the Main Outcome of Sepsis in UK Biobank

Supplement 3. Data Sharing Statement
