# Supplementary material for: Association of Genetically Predicted Insomnia With Risk of Sepsis: A Mendelian Randomization Study
Source: JAMA Psychiatry. 2023 Aug 9;80(10):1061–5. doi: 10.1001/jamapsychiatry.2023.2717 (PMC10413214; doi:10.1001/jamapsychiatry.2023.2717)
Supplement: Supplement 2. — eAppendix. Example Code for the Main Outcome of Sepsis in UK Biobank [file jamapsychiatry-e232717-s002.pdf]

Supplemental Online Content

Thorkildsen MS, Gustad LT, Mohus RM, et al. Association of genetically predicted insomnia with risk of sepsis: a mendelian randomization study. *JAMA Psychiatry*. Published online August 9, 2023. doi:10.1001/jamapsychiatry.2023.2717

eAppendix. Example Code for the Main Outcome of Sepsis in UK Biobank

This supplementary material has been provided by the authors to give readers additional information about their work.

© 2023 Thorkildsen MS et al. *JAMA Psychiatry*.

eAppendix. Example Code for the Main Outcome of Sepsis in UK Biobank

```
#########################
#                 #
# UNIVARIABLE ANALYSIS  #
#                 #
#########################

library(TwoSampleMR)

exposure_dat <- read_exposure_data(
  filename="Insomnia_Watanabe2022_UNIVARIABLE_bothSexes.txt",
  sep = "\t",
  snp_col="rsID",
  beta_col="BETA",
  se_col="SE",
  effect_allele_col = "Allele1",
  pval_col = "p",
  eaf_col = "MAF",
  other_allele_col = "Allele2"
)

exposure_dat<- clump_data(exposure_dat, clump_r2=0.01)

outcome_dat <- extract_outcome_data(
    snps = exposure_dat$SNP,
    outcomes = c('ieu-b-69')
)

dat <- harmonise_data(
  exposure_dat = exposure_dat,
  outcome_dat = outcome_dat,
  action = "1"
)

mr(dat,
method_list=c("mr_ivw","mr_weighted_median","mr_weighted_mode","mr_egger_
regression", "mr_wald_ratio"))


###########################
#                   #
#  MULTIVARIABLE ANALYSIS #
#                   #
###########################

library(MendelianRandomization)

# Load SNPs for exposure and mediators. Load SNPs for outcome. Merge and
harmonize to exposure_outcome_dat dataframe. Then run following code for
multivariable Mendelian randomization and to calculate confidence
intervals for proportion mediated. Example is for all mediators.

# MVMR analysis accounting for all covariates:

mr_mvivw(mr_mvinput(cbind(exposure_outcome_dat$Beta_Ins,
exposure_outcome_dat$Beta_T2DM, exposure_outcome_dat$Beta_BMI,
exposure_outcome_dat$Beta_Smk, exposure_outcome_dat$Beta_CVD),
```

© 2023 Thorkildsen MS et al. *JAMA Psychiatry*.

```
cbind(exposure_outcome_dat$SE_Ins, exposure_outcome_dat$SE_T2DM,
exposure_outcome_dat$SE_BMI, exposure_outcome_dat$SE_Smk,
exposure_outcome_dat$SE_CVD), exposure_outcome_dat$beta.outcome,
exposure_outcome_dat$se.outcome))

# Confidence intervals for proportion mediated when accounting for all
covariates:

bx <- exposure_outcome_dat$Beta_Ins
bxse <- exposure_outcome_dat$SE_Ins
by <- exposure_outcome_dat$beta.outcome
byse <- exposure_outcome_dat$se.outcome
bm1 <- exposure_outcome_dat$Beta_BMI
bmse1 <- exposure_outcome_dat$SE_BMI
bm2 <- exposure_outcome_dat$Beta_Smk
bmse2 <- exposure_outcome_dat$SE_Smk
bm3 <- exposure_outcome_dat$Beta_CVD
bmse3 <- exposure_outcome_dat$SE_CVD
bm4 <- exposure_outcome_dat$Beta_T2DM
bmse4 <- exposure_outcome_dat$SE_T2DM

set.seed(31415)
straps = 1e5; total=NULL; direct=NULL
for (j in 1:straps) {
bx.boot   = rnorm(length(bx), bx, bxse)
bm.boot1   = rnorm(length(bm1), bm1, bmse1)
bm.boot2   = rnorm(length(bm2), bm2, bmse2)
bm.boot3   = rnorm(length(bm3), bm3, bmse3)
bm.boot4   = rnorm(length(bm4), bm4, bmse4)
by.boot   = rnorm(length(by), by, byse)
total[j]  = lm(by.boot~bx.boot-1, weights=byse^-2)$coef[1]
direct[j] = lm(by.boot~bx.boot+bm.boot1+bm.boot2+bm.boot3+bm.boot4-1,
weights=byse^-2)$coef[1]
}
mediated = (total-direct)/total
```

© 2023 Thorkildsen MS et al. *JAMA Psychiatry*.
