# Supplementary material for: Association of Genetically Predicted Insomnia With Risk of Sepsis: A Mendelian Randomization Study
Source: JAMA Psychiatry. 2023 Aug 9;80(10):1061–5. doi: 10.1001/jamapsychiatry.2023.2717 (PMC10413214; doi:10.1001/jamapsychiatry.2023.2717)
Supplement: Supplement 3. — Data Sharing Statement [file jamapsychiatry-e232717-s003.pdf]

Data Sharing Statement

Thorkildsen. Association of Genetically Predicted Insomnia With Risk of Sepsis. *JAMA Psychiatry*. Published August 09, 2023. doi:10.1001/jamapsychiatry.2023.2717

Data
Data available: No

Additional Information
Explanation for why data not available: Data is publicly available from other sources.
